# The Influence of Milling on the Dissolution Performance of Simvastatin

**DOI:** 10.3390/pharmaceutics2040419

**Published:** 2010-12-17

**Authors:** Ulrike Zimper, Jaakko Aaltonen, Karen Krauel-Goellner, Keith C. Gordon, Clare J. Strachan, Thomas Rades

**Affiliations:** 1 Solid State Group, School of Pharmacy, University of Otago, 18 Frederick Street, 9016 Dunedin, New Zealand; E-Mails: ulrike.zimper@otago.ac.nz (U.Z.); clare.j.strachan@otago.ac.nz (C.J.S.); 2 School of Pharmacy, Faculty of Health Sciences, University of Eastern Finland, Yliopistonranta 1C, 70210 Kuopio, Finland; E-Mail: jaakko.aaltonen@uef.fi (J.A.); 3 Pharmaceutical Development, Boehringer Ingelheim Vetmedica GmbH, Binger Str. 173, 55216 Ingelheim am Rhein, Germany; E-Mail: karen.krauel-goellner@boehringer-ingelheim.com (K.K.-G.); 4 Department of Chemistry, MacDiarmid Institute for Advanced Materials and Nanotechnology, University of Otago, Union Place West, 9016 Dunedin, New Zealand; E-Mail: Kgordon@chemistry.otago.ac.nz (K.C.G.)

**Keywords:** dissolution rate, ball milling, process induced disorder, response surface modeling, simvastatin

## Abstract

Particle size reduction is a simple means to enhance the dissolution rate of poorly water soluble BCS-class II and IV drugs. However, the major drawback of this process is the possible introduction of process induced disorder. Drugs with different molecular arrangements may exhibit altered properties such as solubility and dissolution rate and, therefore, process induced solid state modifications need to be monitored. The aim of this study was two-fold: firstly, to investigate the dissolution rates of milled and unmilled simvastatin; and secondly, to screen for the main milling factors, as well as factor interactions in a dry ball milling process using simvastatin as model drug, and to optimize the milling procedure with regard to the opposing responses particle size and process induced disorder by application of a central composite face centered design. Particle size was assessed by scanning electron microscopy (SEM) and image analysis. Process induced disorder was determined by partial least squares (PLS) regression modeling of respective X-ray powder diffractograms (XRPD) and Raman spectra. Valid and significant quadratic models were built. The investigated milling factors were milling frequency, milling time and ball quantity at a set drug load, out of which milling frequency was found to be the most important factor for particle size as well as process induced disorder. Milling frequency and milling time exhibited an interaction effect on the responses. The optimum milling settings using the maximum number of milling balls (60 balls with 4 mm diameter) was determined to be at a milling frequency of 21 Hz and a milling time of 36 min with a resulting primary particle size of 1.4 μm and a process induced disorder of 6.1% (assessed by Raman spectroscopy) and 8.4% (assessed by XRPD), at a set optimization limit of < 2 μm for particle size and < 10% for process induced disorder. This optimum was tested experimentally and the process induced disorder was determined to be 6.9% (± 2.2) by Raman spectroscopy and 7.8% (± 2.3) by XRPD. Subsequent intrinsic dissolution testing revealed that the process induced disorder was negligible with regard to the dissolution rate. The predicted primary particle size of 1.4 μm could be confirmed experimentally, but due to agglomeration of the primary particles a dissolution rate advantage was not shown, highlighting the importance of dissolution testing at an early stage of drug development.

## 1. Introduction

Chemometric methods such as design of experiments (DoE) and multivariate analysis (MA) are suitable methodological approaches for the purpose of optimization and standardization of pharmaceutical unit operations [1,2,3,4]. Moreover, DoE is a powerful technique applied to making experiments more efficient [5]. Several factors are varied simultaneously in a systematic way using the concept of e.g., factorial designs [5]. This approach of experimentation is based on mathematical models, which make it possible to investigate several factors together and still draw safe conclusions about individual effects [5]. In order to improve drug development in the pharmaceutical industry, the FDA (US Food and Drug Administration) is promoting the use of Quality by Design (QbD) and Process Analytical Technologies (PAT), for which DoE and MA are building blocks [6]. The ICH (The International Conference on Harmonization of Technical Requirements for Registration of Pharmaceuticals for Human Use) also outlined the concept of design space in its Q8 (R2) - guideline (“Pharmaceutical Development”) in 2009. This study explores how DoE and MA can be utilized to address a specific problem related to the quality of the final product, in this case, particle size reduction with a minimal impact on the solid state of the drug. Ultimately, the optimized formulation was tested with regard to its dissolution behavior as the decisive criterion for the drug performance. 

Milling is a common diminution technique in the pharmaceutical industry to improve the performance of drugs, including their dissolution rates [7,8]. However, the major drawback of this process is the possible mechanical activation of the milled drug and subsequent introduction of process induced disorder [7]. Process induced disorder in this context is defined as a pre-amorphous solid state, which does not exhibit a glass transition, as opposed to the amorphous state, which is defined by the presence of a glass transition upon thermal treatment [9]. Drugs with different molecular arrangements may exhibit different properties such as solubility and dissolution rate (and hence, possibly bioavailability), necessitating the need to monitor process induced solid state modifications [10,11].

Specifically, this study explores the potential of an optimization design, namely a central composite face centered design, applied to a dry ball milling process. The investigated milling factors were milling frequency, milling time and ball quantity at a set drug load. The impact of these factors on the responses particle size and process induced disorder was explored in the full factorial design as part of the optimization design. Particle size was the main response and process induced disorder the second, adverse, response investigated. Response surface modeling was undertaken and subsequent overlay of response surfaces in the form of optimal area plots determined optimal milling parameters in order to gain the smallest possible particle size while limiting the introduction of process induced disorder. Subsequently, the optimal milling parameters were tested experimentally with regard to the responses particle size and process induced disorder as well as ultimately, the dissolution rate.

## 2. Experimental Section

### 2.1. Material

Simvastatin (M = 418.57 g/mol), (Salutas Pharma, Germany, Batch No. B-710543/00157) was used as received.

### 2.2. Ball milling

Drug samples (600 mg) were placed in 25 mL stainless steel jars and milled using an oscillatory ball mill (Mixer Mill MM301, Retsch GmbH and Co., Germany) at 4 °C. Processing times (5-60 min), milling frequency (5-25 Hz) and number of stainless steel balls (3-60 with a diameter of 4 mm) were selected using a central composite face centered design (MODDE software version 7, Umetrics AB, Sweden), (Table 1). The processed samples were stored over silica gel at 4 °C and investigated by differential scanning calorimetry (DSC), X-ray powder diffraction (XRPD) and Raman spectroscopy within 24 hours after preparation. Particle size was assessed by scanning electron microscopy (SEM) and image analysis.

### 2.3. Experimental design

A central composite face centered design was used for the milling experiments as shown in Table 1. The experimental design was created using MODDE software (version 7, Umetrics AB, Sweden). Response surface modeling led to optimal area plots which were utilized to determine the optimal milling parameters. The quadratic models were fitted to the data using multiple linear regression (MLR) including linear, interaction and square terms. The model significance was assessed using analysis of variance (ANOVA).

### 2.4. Differential scanning calorimetry (DSC)

DSC thermograms (DSC Q100 V8.2 Build 268, TA Instruments, U.S.) were obtained under a nitrogen gas flow of 50 mL/min. Calibration of the DSC instrument was carried out using indium (for temperature and enthalpy) and sapphire (for heat capacity) as standards. Sample powders were crimped in aluminum pans and heated at a scanning rate of 20 K/min from -10 to 200 °C. The thermograms were recorded and analyzed using TA Universal Analysis software (version 4.0 C).

### 2.5. X-ray powder diffraction (XRPD)

XRPD analysis was performed using an X’Pert PRO X-ray diffractometer (PANalytical, The Netherlands; MPD PW3040/60 XRD; CuKα anode; λ = 1.541 Å). The samples were gently consolidated in an aluminum holder and scanned at 40 kV and 30 mA from 5-35°2θ using a scanning speed of 0.1285°/min and a step size of 0.0084°. The diffractograms were collected using X’Pert High Score software (version 2.2.0).

### 2.6. FT-Raman spectroscopy

FT-Raman spectra were obtained using a Bruker IFS 55 FT-Raman interferometer fitted with a Bruker FRA 106 S FT-Raman accessory (Bruker Optik GmbH, Germany). The instrument used a D418-T Ge diode detector and a Coherent Compass 1064-500N laser (Coherent Inc., U.S.). Analysis was carried out at room temperature with the wavelength of the Nd:YAG laser set at 1064 nm and the laser power set at 120 mW. Samples were packed in glass sample holders and spectra were collected at a resolution of 4 cm^-1^. The number of scans obtained per spectrum was 32. Sulfur was used as reference standard to monitor wavenumber accuracy. Data were collected using OPUS^TM^-software (Bruker Optik GmbH, Germany).

### 2.7. Multivariate analysis

Partial least squares regression (PLS) analysis (SIMCA-P 11 software, Umetrics AB, Sweden) was used to analyze the XRPD diffractograms and Raman spectra. Whole diffractograms (5 to 35°2θ) and spectra (500 to 3500 cm^-1^) were used for the analysis. PLS models for the XRPD data were computed using orthogonal signal correction (OSC) and mean centering as preprocessing and scaling methods. For the Raman spectroscopic data, standard normal variate transformation (SNV) was applied as a preprocessing method and the spectra were mean centered prior to modeling [9]. 

### 2.8. Scanning electron microscopy (SEM)

**Figure 1:**
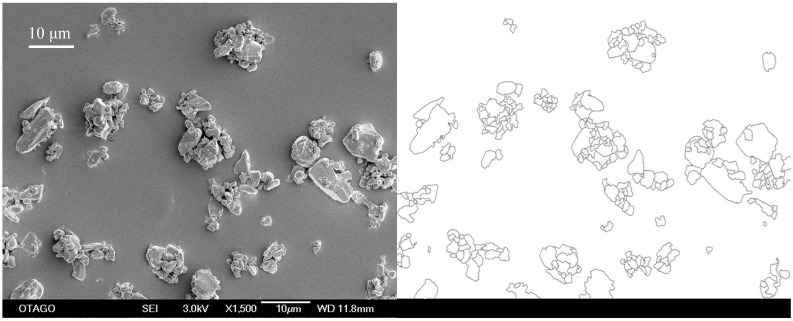
SEM-image of unprocessed simvastatin as received (left; bar equals 10 μm) and the edited image used for particle size analysis (right; particles on the border of the image were excluded). For particle size assessment, the evaluation axis was varied in 1° –steps from 0º to 180º and the particle diameter determined at each angle. The arithmetic mean of all diameters gave the mean diameter.

SEM was performed using a JEOL JSM-6700F field emission scanning electron microscope (JEOL Ltd, Tokyo, Japan). Samples were sputter coated with a 15 nm gold palladium layer in an Emitech K575X Peltier-cooled high resolution sputter coater (EM Technologies Ltd, Kent, England) and thereafter analyzed at a suitable working distance (15-3 mm) and magnification (140-60,000×). The acceleration voltage was 3 kV. The images were analyzed with AnalySIS image analysis software (Soft Imaging System GmbH, Münster, Germany) and a minimum of 400 particles per sample was used for particle size analysis (Figure 1). The arithmetic mean of all diameters of a particle (for evaluation axis angles 1°, 2°, 3°, …, 180°), *i.e*., the mean diameter was used for the determination of the primary particle size. Finally, the particle size was determined in terms of D100, *i.e*, the biggest detectable particle size for the respective sample was decisive.

### 2.9. Solubility study

The solubility of simvastatin was determined in triplicate at 37 °C in pH 7 buffer solution containing 0.25% sodium dodecyl sulfate (SDS) in 0.01 M sodium phosphate (15 g SDS and 8.28 g monobasic sodium phosphate in 6,000 mL of Milli-Q water, adjusted with 5% (w/v) sodium hydroxide solution to a pH of 7.0 [12,13]. Simvastatin was put in excess into the dissolution medium and agitated for 24 hours. A standard curve was established for a concentration range of 2.5 to 15 μg/mL (r^2^ = 0.999) by UV spectrometry at a wavelength of 238 nm (Ultrospec 2000, Pharmacia Biotech, Cambridge, UK).

### 2.10. Dissolution test

The dissolution method applied was adapted from the USP 2009, Pandya *et al.* and Singla *et al.* [12,13,14]. Intrinsic and powder dissolution tests were undertaken for the processed as well as unprocessed compound. For the intrinsic dissolution test, 250 mg of pure compound were directly pressed into a disk at 2 t (Carver Inc., Wisconsin, U.S.). The disks were placed into a PTFE sample holder with only one face exposed to the dissolution medium and dissolution was determined for 30 minutes at 37 °C in 900 mL phosphate buffer pH 7 containing 0.25% SDS, at a rotation speed of 50 rpm. The powder dissolution test was undertaken with the same parameters under sink conditions, *i.e*., 40 mg compound were used and the paddle method according to the USP monograph for simvastatin was applied [14]. Sample aliquots of 5 mL were taken after 2, 5, 10, 15, 20, 25 and 30 minutes and the dissolution medium was replaced immediately. The UV spectrometer was set at 238 nm. All samples were filtered through 0.45 μm filters (mixed cellulose ester filters, ADVANTEC, Toyo Roshi Kaisha, Ltd., Japan) prior to measurement. One way analysis of variance was performed with Minitab software (Minitab, State College PA, U.S.).

## 3. Results and Discussion

The central composite face centered design including the respective response values is shown in Table 1. PLS models for the Raman spectroscopic data as well as for the XRPD data were utilized in order to acquire the values for process induced disorder. The models were explained in detail previously [9].

**Table 1:** Central composite face centered design showing all particular milling settings as well as the respective response values. Experiments No. 15 to 17 are the center point experiments.

No.	Time Minutes	Frequency Hertz	No. of balls 4 mm	Primary particle size D 100 μm	% Process induced disorder Raman	% Process induced disorder XRPD
1	5	5	3	11.8	0.4	0.5
2	60	5	3	12.8	0.4	0.2
3	5	25	3	11.9	2.2	-2.4
4	60	25	3	1.1	7.5	10.7
5	5	5	60	12.8	-0.4	1.4
6	60	5	60	12.1	0.2	0.7
7	5	25	60	3.3	3.7	8
8	60	25	60	0.3	15.5	18.3
9	5	15	31	12.8	1.4	-1.1
10	60	15	31	4.5	3.2	2.1
11	32.5	5	31	9.3	0.2	-0.6
12	32.5	25	31	1.1	7.3	7.9
13	32.5	15	3	12.1	0	0.5
14	32.5	15	60	2.2	1.9	2.2
15	32.5	15	31	11.3	1.7	0.7
16	32.5	15	31	5	1	-3.8
17	32.5	15	31	12.8	1.7	1.7

**Figure 2:**
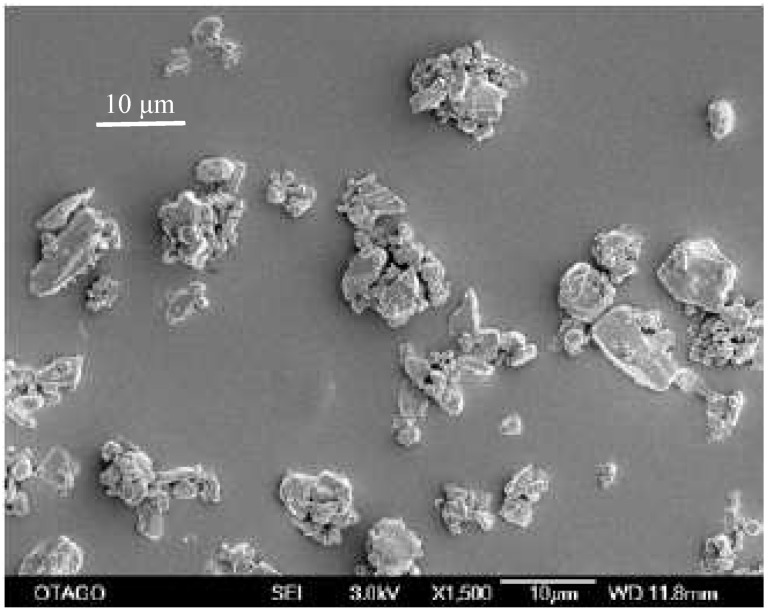
SEM-image of unprocessed simvastatin as received. The bar equals 10 μm.

Scanning electron microscopy was chosen as the method to determine the particle size as the milling operation was undertaken under dry conditions with resulting agglomerated particles. The initial primary particle size of the unprocessed compound was determined to be 12.8 μm (Figure 2).

**Figure 3:**
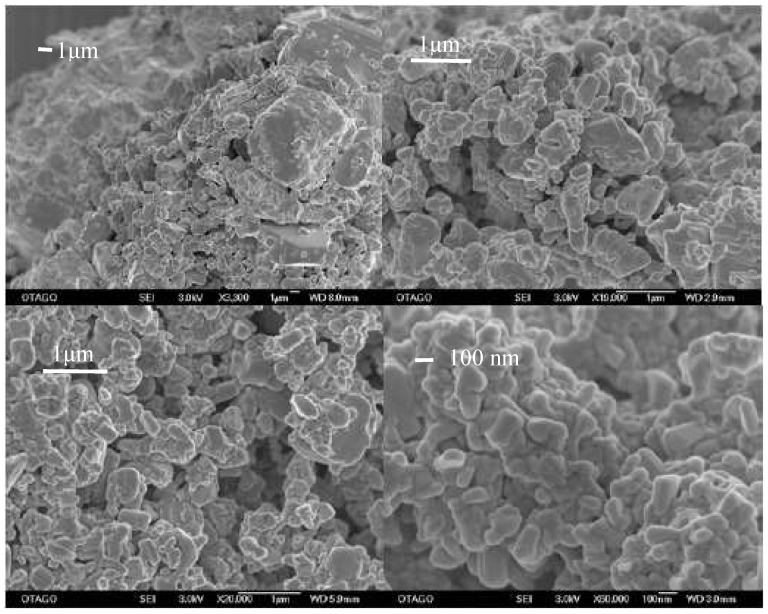
Images of sample 3 (tope left), 4 (top right), sample 12 (bottom left) and 8 (bottom right). Sample 8 is the nano-crystalline sample. Bars equal 1 μm, except for the nano-crystalline sample, where the bar equals 100 nm.

It could be shown that for all samples milled at the high milling frequency (25 Hz), a micro- and nano-particulate fraction were obtained (Figure 3). However, only for the sample milled at the highest milling frequency (25 Hz) and longest milling time (60 min) with the maximum number of 60 milling balls, a completely nano-particulate sample was achieved (Figure 3).

**Figure 4:**
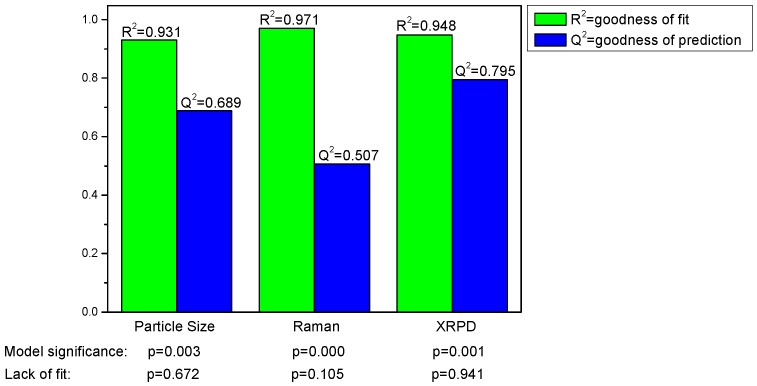
Model parameters for the responses particle size and process induced disorder, assessed by Raman spectroscopy and X-ray powder diffraction (XRPD), respectively.

Valid and significant quadratic models were built. The model parameters R^2^ (goodness of fit) and Q^2^ (goodness of prediction) are shown in Figure 4 and reveal that all models are valid. The models were significant according to ANOVA with no lack of fit and the respective p-values are also listed in Figure 4.

For the models presented, the model parameters suggest a better prediction of process induced disorder for the model based on the XRPD data.

**Figure 5:**
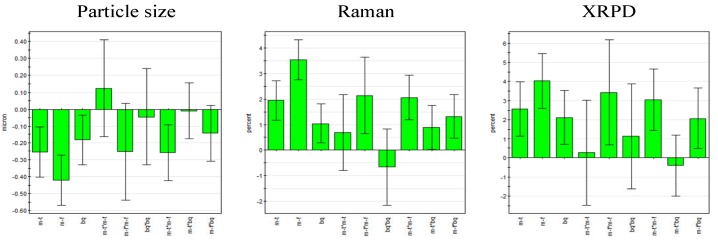
Regression coefficient plot of the scaled and centered coefficients for particle size (left) and process induced disorder determined by Raman spectroscopy (center) and XRPD (right) (error bars represent 95% confidence interval; m-f = milling frequency, m-t = milling time, bq = ball quantity).

According to the regression coefficient plots, all three linear terms (milling frequency, milling time and ball quantity) were statistically significant and milling frequency was found to be the most important factor for all responses. Furthermore, it was found that milling frequency and milling time exhibited an interaction effect on the responses. Milling frequency was the only statistically significant square term (Figure 5). No term was omitted as model improvement was negligible. 

**Figure 6:**
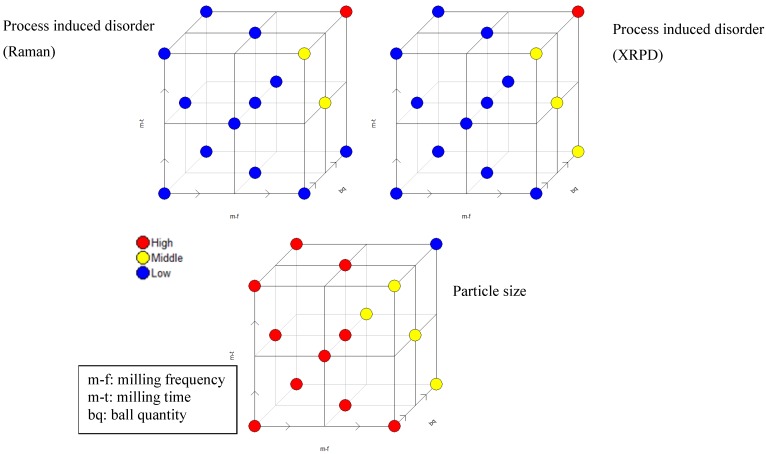
Central composite face centered design for simvastatin based on process induced disorder assessed by Raman spectroscopy (upper left) and XRPD (upper right) and particle size (bottom) as respective responses. “High” (red), “Middle” (yellow) and “Low” (blue) refer to the effect on the respective response.

The full factorial design as a screening tool is part of the central composite design (optimization design), (Figure 6). The colors symbolize the effect on the respective responses at the respective milling setting; each point symbolizes one particular experiment according to Table 1. Figure 5 and Figure 6 visualize that particle size and process induced disorder are opposing responses with respect to variable effect.

**Figure 7:**
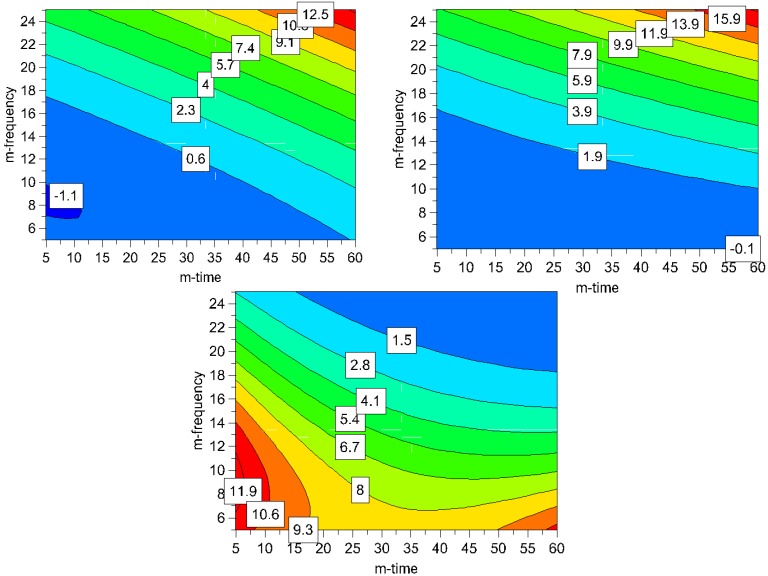
Contour plots for process induced disorder [%] assessed by Raman spectroscopy (top left) and XRPD (top right) and particle size [μm] (bottom); the ball quantity was set high (60 balls) for all plots to assure a high media-drug interaction leading to smaller particles (m-frequency = milling frequency; m-time = milling time).

**Figure 8:**
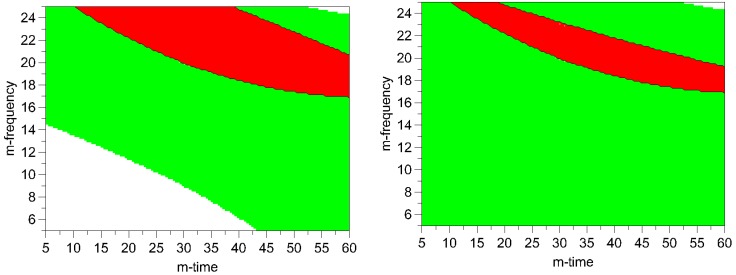
Optimal area plots for particle size and process induced disorder assessed by Raman spectroscopy (left) and XRPD (right). Optimization limits were set at < 2 μm for particle size and < 10% for process induced disorder at a high ball quantity of 60 balls. The optimal area is shown in red (m-frequency = milling- frequency; m- time = milling- time). Green areas indicate the experimental space where one of the limiting conditions is met. The white areas indicate where none of the limits are met.

It can be seen in the contour plots for process induced disorder, that the lowest impact on process induced disorder can be achieved by applying low to middle milling frequencies (5-17 Hz) over the whole time frame (5-60 min), (Figure 7). However, particle size in this operating space yields bigger particles (8-11.9 μm). Hence, the optimum was determined by overlaying the respective contour plots and, thus, creating the optimal area plots, and the results are shown in Figure 8. The optimization limits were set at < 2 μm for particle size and < 10% for process induced disorder with a small particle size as the decisive criterion. A high ball quantity of 60 balls was chosen to ensure sufficient interaction of the milling media with the drug, as the main objective was to reduce the particle size. Optimal milling parameters for simvastatin were found to be at high and middle milling frequencies and middle milling times. In this experimental space, the local optimum according to the model could be found at a milling frequency of 21 Hz and 36 min milling time (ball quantity 60). These milling conditions resulted in a predicted primary particle size of 1.4 μm and a prediction of process induced disorder of 6.1% (when determined by Raman spectroscopy) and 8.4% (when determined by XRPD).

**Figure 9:**
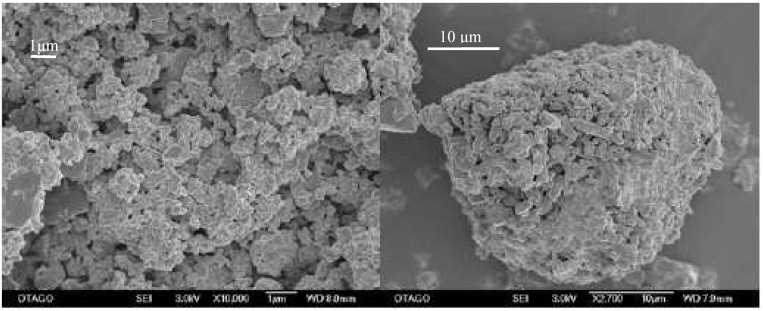
Primary (left) and secondary (right) particle size of the optimized milled sample. Bars equal 1 and 10 μm, respectively.

Subsequently, the predicted optimum was tested experimentally and a process induced disorder of 6.9% (± 2.2) was determined by Raman spectroscopy and 7.8% (± 2.3) was determined by XRPD, which complies with the prediction. No glass transition could be detected in any of the samples by DSC, thus amorphization of the drug was excluded. The predicted primary particle size of 1.4 μm could also be confirmed experimentally. However, the high surface energy of the small particles resulted in the formation of large agglomerated secondary particles, which proved to be of significance for the dissolution performance (Figure 9).

**Figure 10:**
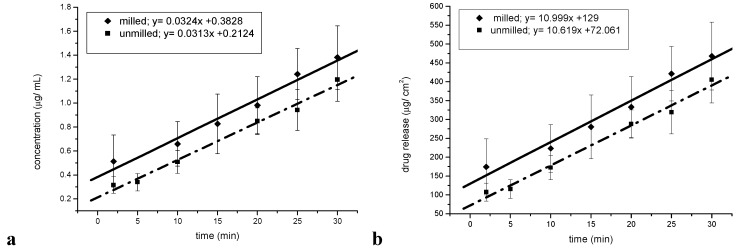
Intrinsic dissolution tests. (**a**) Concentration-time profile for the intrinsic dissolution of milled and unmilled simvastatin. (**b**) Release-time profile for the intrinsic dissolution of milled and unmilled simvastatin.

Intrinsic and powder dissolution tests were undertaken in order to test whether the reduced particle size in fact leads to a dissolution rate enhancement. The saturation solubility of unprocessed simvastatin in the dissolution medium was found to be 0.51 mg/ mL (± 0.030), which is in accordance with published values [12]. The concentration-time profiles (mg/mL) and the release-time profiles (mg/cm^2^) for the intrinsic dissolution of the milled and unmilled simvastatin are shown in Figure 10a and 10b. The surface area of the pure sample tablets was 2.65 cm^2^, and the intrinsic dissolution rates (IDRs) of the milled and unmilled simvastatin were 11.00 μg/cm^2^ min and 10.62 μg/cm^2^ min, respectively (Figure 10b). The graphs of the IDR plots were parallel and the difference was not statistically significant (α = 0.05, P = 0.421; n = 3), indicating that the process induced disorder was negligible with regard to the dissolution rate.

**Figure 11:**
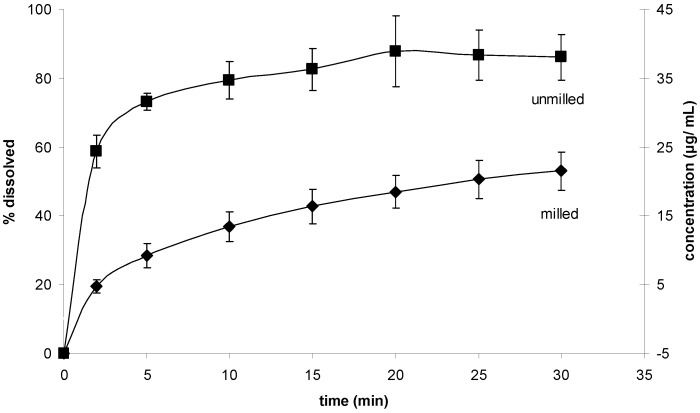
Powder dissolution test. Dissolution rates of milled and unmilled simvastatin.

The results for the powder dissolution tests are shown in Figure 11. Interestingly, the unprocessed sample showed the higher dissolution rate.

Since the investigated milling procedure was a dry milling process in the absence of stabilizing excipients, agglomeration is likely to be accountable for the poor dissolution performance of the processed drug compared to the unprocessed drug. Wet milling of the drug in a media mill may be a feasible alternative, as in this milling operation small particles are prevented from agglomeration through steric and/ or electrostatic stabilization [15].

## 4. Conclusions

A central composite face centered design was successfully applied to determine optimum milling parameters for simvastatin in a dry ball milling process with regard to particle size and process induced disorder. Intrinsic dissolution testing showed the process induced disorder to be insignificant with regard to the dissolution rate. However, due to agglomeration, the primary particle size advantage was not translated into a dissolution rate advantage. This study shows the importance of dissolution testing at an early stage of drug development in order to ensure the performance of the final drug formulation.
